# Comparison of fast multi-slice and standard segmented techniques for detection of late gadolinium enhancement in ischemic and non-ischemic cardiomyopathy – a prospective clinical cardiovascular magnetic resonance trial

**DOI:** 10.1186/s12968-018-0434-2

**Published:** 2018-02-19

**Authors:** Fabian Muehlberg, Kristin Arnhold, Simone Fritschi, Stephanie Funk, Marcel Prothmann, Josephine Kermer, Leonora Zange, Florian von Knobelsdorff-Brenkenhoff, Jeanette Schulz-Menger

**Affiliations:** 1 Working Group on Cardiovascular Magnetic Resonance, Experimental and Clinical Research Center - a joint cooperation between the Charité Medical Faculty and the Max-Delbrück Center for Molecular Medicine and HELIOS Hospital Berlin-Buch, Department of Cardiology and Nephrology, Lindenberger Weg 80, 13125 Berlin, Germany; 2 Clinic Agatharied, Department of Cardiology, Ludwig-Maximilians-University Munich, Norbert-Kerkel-Platz, 83734, Hausham, Germany

**Keywords:** Cardiac MR, CMR, Late gadolinium enhancement, Single-shot, Hypertrophic cardiomyopathy, Myocardial infarction, Inflammatory heart disease, Myocarditis

## Abstract

**Background:**

Segmented phase-sensitive inversion recovery (PSIR) cardiovascular magnetic resonance (CMR) sequences are reference standard for non-invasive evaluation of myocardial fibrosis using late gadolinium enhancement (LGE). Several multi-slice LGE sequences have been introduced for faster acquisition in patients with arrhythmia and insufficient breathhold capability.

The aim of this study was to assess the accuracy of several multi-slice LGE sequences to detect and quantify myocardial fibrosis in patients with ischemic and non-ischemic myocardial disease.

**Methods:**

Patients with known or suspected LGE due to chronic infarction, inflammatory myocardial disease and hypertrophic cardiomyopathy (HCM) were prospectively recruited. LGE images were acquired 10–20 min after administration of 0.2 mmol/kg gadolinium-based contrast agent. Three different LGE sequences were acquired: a segmented, single-slice/single-breath-hold fast low angle shot PSIR sequence (FLASH-PSIR), a multi-slice balanced steady-state free precession inversion recovery sequence (bSSFP-IR) and a multi-slice bSSFP-PSIR sequence during breathhold and free breathing. Image quality was evaluated with a 4-point scoring system. Contrast-to-noise ratios (CNR) and acquisition time were evaluated. LGE was quantitatively assessed using a semi-automated threshold method. Differences in size of fibrosis were analyzed using Bland-Altman analysis.

**Results:**

Three hundred twelve patients were enrolled (*n* = 212 chronic infarction, *n* = 47 inflammatory myocardial disease, *n* = 53 HCM) Of which 201 patients (67,4%) had detectable LGE (*n* = 143 with chronic infarction, *n* = 27 with inflammatory heart disease and *n* = 31 with HCM). Image quality and CNR were best on multi-slice bSSFP-PSIR. Acquisition times were significantly shorter for all multi-slice sequences (bSSFP-IR: 23.4 ± 7.2 s; bSSFP-PSIR: 21.9 ± 6.4 s) as compared to FLASH-PSIR (361.5 ± 95.33 s). There was no significant difference of mean LGE size for all sequences in all study groups (FLASH-PSIR: 8.96 ± 10.64 g; bSSFP-IR: 8.69 ± 10.75 g; bSSFP-PSIR: 9.05 ± 10.84 g; bSSFP-PSIR free breathing: 8.85 ± 10.71 g, *p* > 0.05).

LGE size was not affected by arrhythmia or absence of breathhold on multi-slice LGE sequences.

**Conclusions:**

Fast multi-slice and standard segmented LGE sequences are equivalent techniques for the assessment of myocardial fibrosis, independent of an ischemic or non-ischemic etiology. Even in patients with arrhythmia and insufficient breathhold capability, multi-slice sequences yield excellent image quality at significantly reduced scan time and may be used as standard LGE approach.

**Trial registration:**

ISRCTN48802295 (retrospectively registered).

## Background

Late gadolinium enhancement (LGE) cardiovascular magnetic resonance (CMR) is a well-established method for assessment of focal myocardial fibrosis and scarring in ischemic and non-ischemic cardiomyopathies [1–4]. The presence and extent of LGE has been shown to be associated with worse patient outcome in a variety of diseases, i.e. myocardial infarction, hypertrophic cardiomyopathy (HCM) and acute or chronic inflammatory heart disease [5–7]. Hence, the assessment of LGE is integrated into many clinical guidelines and is an integral part of most contrast-based CMR protocols [8–10].

The reference standard technique for LGE assessment is typically based on phase-sensitive inversion recovery (PSIR) sequences that are acquired in a single-slice, single-breathhold fashion [11, 12]. These segmented PSIR LGE images generate excellent image quality at a high spatial resolution if the individual patient has sufficient breathhold capabilities and is in sinus rhythm [13].

However, with the more widespread use of CMR in clinical routine increasing numbers of patients referred for CMR present with arrhythmias or an inability for sufficient breathhold for CMR scan. In these patients, conservative segmented PSIR LGE sequences sometimes fail to provide satisfactory image quality for accurate assessment.

Furthermore, standard segmented LGE sequences typically require 5 to 10 min of scan time for complete myocardial coverage. There is a need for faster and more efficient imaging in CMR in order to enable a more wide-spread use of CMR in clinical routine as well as in smaller institutions where access to CMR scanners maybe more restricted [14]. CMR also competes with other non-invasive imaging techniques in terms of scan time optimization leading to efforts for faster standardized CMR scan protocols [15].

In order to address these issues, multi-slice LGE sequences have been developed with acquisition of the entire k-space of an individual image slice within one heart cycle [16]. Different approaches utilize navigator-based, free breathing sequencing which works without breathhold but mostly still requires stable heart rhythm for optimal image quality [17].

Several small clinical studies have shown that multi-slice LGE sequences provide similar image quality to standard segmented LGE sequences [18, 19]. However, the vast majority of these studies investigated only patients with a single disease entity, i.e. myocardial infarction or HCM and/or excluded patients with arrhythmia. Hence, these studies are not reflecting clinical reality where the underlying cause of LGE is often not known prior to the CMR scan and sinus rhythm is often unstable or non-existent.

In this prospective study we intended to determine the comparability of standard segmented PSIR LGE imaging with two different multi-slice LGE sequences with and without breathhold in a large number of patients with ischemic and non-ischemic cardiomyopathy, namely chronic myocardial infarction, HCM and inflammatory heart disease. Furthermore, we explicitly did not exclude patients with arrhythmia. We aimed to assess if multi-slice LGE sequences represent a robust alternative for LGE assessment independent of pathophysiologic origin of LGE, heart rhythm and patient breathhold capabilities.

## Methods

### Study population

312 consecutive patients with known or suspected LGE were prospectively recruited. All patients were referred for clinical LGE assessment using CMR for both, ischemic and non-ischemic cardiomyopathies, based on the clinical information provided by the referring cardiologist. A total of 212 patients had chronic myocardial infarction, 53 patients had HCM and 47 patients had inflammatory heart disease.

All patients underwent a single CMR scan with three different LGE sequences. Exclusion criteria were contraindications to CMR and severe chronic renal disease with an estimated glomerular filtration rate < 30 ml/min/1.73m^2^. All studies were performed in accordance with the local institutional review board and local ethics committee approval.

### Image protocol

All CMR studies were performed on a 1.5 Tesla scanner (AvantoFit®, Siemens Healthineers, Erlangen, Germany). Patients were scanned with electrocardiogram (ECG)-triggering in the supine position using 16-channel surface phased array coils.

All imaging protocols included assessment of myocardial function in balanced steady-state free precession (bSSFP) cine sequences and of myocardial morphology by LGE imaging.

bSSFP cine imaging (TE 1.19 ms, TR 33.36 ms, flip angle 55°, retrospective ECG-triggered gating, matrix 192x156mm, FOV 340 mm, slice thickness 6 mm, bandwidth 930 Hz, 30 phases per heart cycle, iPAT GRAPPA acceleration factor 2) was performed in long axis two- and four-chamber view for biplanar assessment of left ventricular (LV) end-diastolic volume (LVEDV), LV mass (LVM) and LV ejection fraction (LVEF). Contours were drawn manually and biplanar anatomical and functional parameters calculated automatically by the post-processing software according to an established in-line biplane ellipsoid model. [20] The standard three-point method was used on short axis localizers to define standardized long axis two-chamber (one point in the LV apex, one point in the anterior and one point in the inferior wall of the lv myocardium in the slice with the maximum LV area) and four-chamber view (one point in the LV apex, one point in the interatrial septum below the aorta and one point into the most lateral corner of the right ventricle (RV) on the short axis localizer with the maximum RV area).

For LGE imaging, a 0.2 mmol/kg intravenous injection of contrast agent was administered into an antecubital vein. In patients with myocardial infarction or HCM assessment gadoteridol (ProHance®, Bracco S.p.A., Milan, Italy) was used. For patient with known or suspected inflammatory heart disease gadopentetate (Magnevist®, Bayer Healthcare, Wayne, New Jersey, USA) was administered due to established normal values for this contrast agent for the early enhancement technique which was clinically assessed in these patients independently from this study [21].

Ten minutes after contrast administration, a segmented IR cine bSSFP inversion time (TI) scouting sequence was performed at a mid-ventricular short axis location to determine optimal TI [22]. TI was adapted to optimally null the signal of the remote myocardium. Two-dimensional LGE images were acquired in short-axis views covering the entire LV myocardium by using three different LGE sequences: i) a segmented, single-slice, single-breathhold 2D FLASH-based phase-sensitive inversion recovery sequence (FLASH-PSIR) which was considered as the reference standard; ii) a multi-slice 2D bSSFP-based inversion recovery sequence (bSSFP-IR); iii) a multi-slice 2D bSSFP-based PSIR sequence (bSSFP-PSIR).

Sequence details are displayed in Table 1.

**Table 1:** LGE sequence parameters

		FLASH-PSIR	SSFP-IR	SSFP-PSIR
Mode		Segmented	Multi-slice (single shot)	Multi-slice (single shot)
TE	[ms]	5.17	1.06	1.05
Flip Angle		30°	50°	65°
Field of view	[mm]	350–450	350–450	350–450
Matrix	[mm]	192 × 256	154 × 192	144 × 192
Slice thickness	[mm]	7	7	7
Slice gap	[mm]	0	0	0

*TE* Echo time

All LGE sequences were acquired in end-expiratory breathhold while the bSSFP-PSIR sequence was additionally acquired in free breathing. In case of suspected artifacts in the LGE images a second perpendicular slice through the affected region was acquired or read-out of the phase encoding direction was swapped. Segmented and multi-slice LGE images were acquired in random order. Acquisition times and occurrence of arrhythmia during image acquisition were noted for all sequences.

### Qualitative and quantitative image analysis

For all post-processing analyses commercially available software was used (CVI42 Release 5.6.2, Circle Cardiovascular Imaging, Calgary, Canada). A blinded reader performed LV function assessment in bSSFP cine long axis slices. For assessment of LVEF endocardial contours were drawn in the end-diastolic and end-systolic phase of two- and four-chamber view. All parameters were automatically calculated after contouring by the post-processing software. Separately, image quality and quantitative LGE assessment were performed in a random and blinded order.

For 30 randomly selected individuals, the same reader and a second experienced reader repeated analyses for assessment of intra- and interobserver variability.

#### Image quality

Visual assessment of image quality was performed on all LGE sequences for each patient using a previously established 4-point-scale using the following grading: excellent quality, no artifacts (score of 1); good quality, minimal artifacts (score of 2); moderate quality, some artifacts which may impair diagnostic quality (score of 3); poor quality, unacceptable artifacts (score of 4) [19].

Signal intensities were measured in regions of interest (ROIs) that covered areas of contrast-enhanced myocardium, as well as areas of remote non-enhanced myocardium with an additional ROI located outside the patient for calculation of background noise. Signal enhancement was measured as recommended by Simonetti et al. [12].

In detail, we calculated signal intensities and their standard deviations in ROIs of LGE-positive myocardium, as well as areas of remote myocardium. Contrast was defined as difference between mean signal intensity of both ROIs. Image noise was defined as the standard deviation of the signal intensity in the normal-appearing myocardium ROI. Contrast-to-noise ratios (CNR) were calculated by using these values. Measurement of signal-to-noise ratios (SNR) is limited on PSIR images conventionally because the measurement of background noise is invalid in the reconstructed images [23]. Therefore, we did not perform SNR assessment.

### Visual LGE assessment

The distribution area and transmurality of fibrosis was evaluated according to the American Heart Association (AHA) 16-segment model. The distribution area of scar in each segment was scored by the proportion of scar to each segment (0: no LGE, 1: 1–25%, 2: 26–50%, 3: 51–75%, 4: 76–100%).

For each subject, the number of segments with presence or absence of fibrosis and location within the myocardial wall (subendocardial, intramural, subepicardial, transmural) was noted for each LGE sequence as previously described [24].

### Quantitative LGE assessment

Quantification of LGE was performed with the established semi-automated signal threshold versus reference mean (STRM) method as published previously by our and other groups [21, 25, 26]. On all LGE images, endocardial and epicardial contours were manually traced and ROIs were defined in hyperenhanced and remote myocardium.

True LGE was defined by myocardial signal intensity plus 6 standard deviations (SD) above that of remote, normal-appearing myocardium within the same slice in patients with myocardial infarction. For subjects with HCM and inflammatory heart disease, plus 3 SDs were defined as true LGE [27].

The automated LGE detection could be manually corrected by the reader for a specific location to exclude obvious artifacts. After segmentation, myocardial and scar tissue mass (in grams) were calculated and compared for each AHA segment in each sequence.

### Statistical analysis

All statistical analyses were conducted by using statistical software package SPSS 17.0 (International Business Machines, Armonk, New York, USA). Quantitative data are expressed as means ± SD. Sample size was calculated by using power analysis for two proportions to reach a statistical power of more than 80% to detect differences of 5%, using the assumption of 16 ± 12 g scar tissue for patients with chronic myocardial infarction, and 9 ± 5 g for HCM and inflammatory heart disease which were reported previously by our group [26].

Image quality scores were compared by using the Mann-Whitney U test. Interobserver and intraobserver agreement was assessed by using Cohen k statistics.

Statistical comparison of means of LGE size in each individual multi-slice technique against the segmented reference standard technique was performed by using two-tailed paired t tests and Bland-Altman analysis. Scar tissue percentages per segment, CNR and signal enhancement ratios were assessed using the Wilcoxon signed rank test, as these values did not show normal distribution.

## Results

### Patient characteristics

**Fig. 1:**
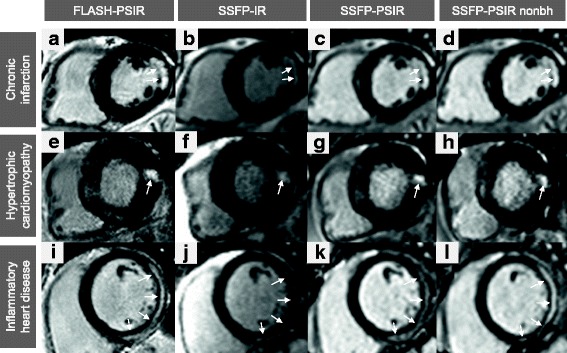
Representative LGE images. Three selected patients with chronic myocardial infarction (**a**-**d**), hypertrophic cardiomyopathy (**e**-**h**) and acute myocarditis (**i**-**l**) with typical LGE localization: subendocardial for infarction, patchy intramural for HCM and subepicardial for myocarditis. Horizontal rows display corresponding slices of LGE in the same patient, vertical columns show the used techniques: conventional segmented FLASH-PSIR (**a**;**e**;**i**), multi-slice bSSFP-IR (**b**;**f**;**j**), multi-slice bSSFP-PSIR with breathhold (**c**;**g**;**k**) and free-breathing multi-slice bSSFP-IR (**d**;**h**;**l**). nonbh = non-breathhold

In total, 312 patients were recruited. Fourteen of these patients were excluded due to incomplete image acquisition. All remaining 298 patients were successfully scanned using all techniques and were included in subsequent analyses (203 patients with chronic myocardial infarction, 50 patients with HCM and 45 patients with inflammatory heart disease). Patient characteristics are shown in Table 2. Study individuals with inflammatory heart disease were significantly younger than patients in the other groups. HCM patients had an increased LVM index (LVM-I), decreased LVEDV index (LVEDV-I) and a slightly elevated LVEF. Figure 1 shows representative images of LGE short axis slices for each group and each sequence.

**Table 2:** Patient Characteristics

	Chronic myocardial infarction	HCM	Inflammatory heart disease
Number of patients	203	50	45
Gender [♂ / ♀]	160 / 43 (78% / 22%)	35 / 15 (72% / 28%)	32 / 13 (71% / 29%)
Age [years]	66.2 ± 10.7	62.0 ± 14.5	46.3 ± 15.4 *
BMI [kg/m2]	27.6 ± 4.2	27.9 ± 4.3	25.8 ± 4.8
HR [min-1]	68.1 ± 11.5	69.8 ± 16.2	72.2 ± 12.9
LVEF [%]	52.9 ± 10.7	63.0 ± 10.9 *	52.6 ± 13.3
LVEDV-I [ml/m^2^]	82.5 ± 24.3	69.6 ± 22.1 *	90.9 ± 26.9
SV-I [ml/m^2^]	41.9 ± 8.7	43.3 ± 12.6	44.8 ± 8.9
LVM-I [g/m^2^]	59.3 ± 15.8	89.5 ± 28.4 *	61.9 ± 17.2
SR / Arrhythmia	166 / 37 (82% / 18%)	39 / 11 (78% / 22%)	38 / 7 (84% / 16%)
LGE detected [yes / no]	176 / 27 (87% / 13%)	39 / 11 (78% / 22%)	32 / 13 (71% / 29%)

*HCM* Hypertrophic cardiomyopathy; *BMI* Body mass index; *HR* Heart rate; *LVEF* Left ventricular ejection fraction; *LVEDV-I* Left ventricular end-diastolic volume index; *SV-I* Stroke volume index; *LVM-I* Left ventricular mass index. *SR* Sinus rhythm. * p < 0.05

### Acquisition time

The average scan time was significantly longer for the reference standard sequence (361.5 ± 95.3 s including breaks between slice acquisitions) than for any multi-slice sequence (SSFP-IR: 23.4 ± 7.2 s; SSFP-PSIR: 21.9 ± 6.4 s, *p* < 0.01 of all sequences against reference standard).

### Image quality assessment

**Fig. 2:**
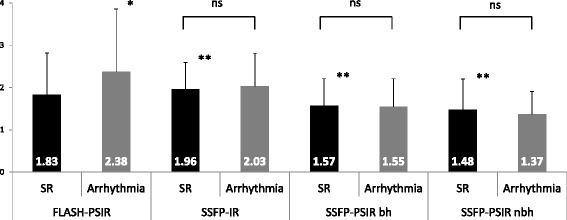
Image quality scores. Values represent average image quality score for all patients in each group. Score system: 1 = excellent quality, no artifacts; 2 = good quality, minimal artifacts; 3 = moderate quality, some artifacts which may impair diagnostic quality; 4 = poor quality, unacceptable artifacts. * *p* < 0.05 within sequence. ** p < 0.05 towards FLASH-PSIR. ns = non-significant, *p* > 0.05. SR = sinus rhythm

Image quality scores differed significantly between each multi-slice and the reference standard sequence. However, they were not influenced by disease entity or – regarding bSSFP-PSIR sequence – breathhold versus free breathing acquisition. Overall, bSSFP-PSIR images showed the best image quality scores. (Fig. 2).

Arrhythmia had a negative impact on image quality scores on the segmented FLASH-PSIR sequence, resulting in poor or non-diagnostic image quality in 48,8% of all patients. Image quality score was not influenced by arrhythmia in any multi-slice sequence.

Assessment of infarcted-to-remote area CNR is shown in Table 3. Mean infarcted-to-remote myocardium CNR was significantly higher on bSSFP-PSIR than on reference standard sequences (*p* < 0.01), and on bSSFP-IR lower than reference standard (p < 0.01). Free breathing acquisition of bSSFP-PSIR slightly decreased mean infarcted-to-remote area CNR as compared to acquisition under breathhold, however, was still superior to reference standard (p < 0.01). LGE due to chronic infarction showed significantly higher CNR values in all sequences than LGE due to HCM or inflammatory heart disease (p < 0.01).

**Table 3:** Contrast-to-noise ratios

	All groups	Chronic infarction	HCM	Inflammatory heart disease
FLASH-PSIR	65.9 ± 71.9	67.9 ± 58.5 *	80.4 ± 126.8	37.0 ± 21.3 *
SSFP-IR	40.1 ± 26.8†	43.2 ± 28.4 *†	38.5 ± 19.9 †	31.5 ± 22.2†
SSFP-PSIR	137.8 ± 103.7†	149.8 ± 114.9 *†	118.4 ± 66.9†	95.7 ± 49.2 *†
SSFP-PSIR nonbh	125.9 ± 72.5†	134.5 ± 72.5 *†	101.7 ± 65.8†	109.0 ± 73.4 †

*p < 0.05 towards the other disease entities for the individual LGE sequence. † p < 0.05 towards FLASH-PSIR gold standard for individual disease entity

### Qualitative LGE analysis

Using reference standard sequence, 201 patients (67.4%) had detectable LGE (*n* = 143 with chronic infarction, *n* = 31 with HCM and *n* = 27 with inflammatory heart disease). All 201 LGE-positive patients also had detectable LGE on bSSFP-IR. With both bSSFP-PSIR sequences, two small LGE lesions (< 1 g scar size) were visually not detected in one patient with chronic infarction and one patient with HCM by two blinded readers.

**Fig. 3:**
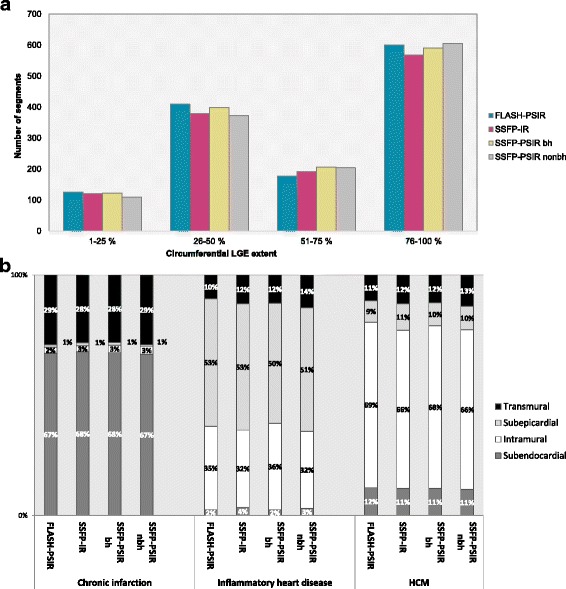
Visual assessment of LGE. **a**: Visual assessment of circumferential LGE extent. Columns represent number of segments with LGE for different circumferential extents across all study groups (chronic myocardial infarctions, HCM, inflammatory heart disease). **b**: Visual assessment of in-wall LGE location

On visual assessment, circumferential extent of scars was similar in all sequences; summation of scores showed excellent matching with reference standard FLASH-PSIR sequence (total score 3875) for bSSFP-PSIR with (total score 3903) and without breathhold (total score 3886) while on bSSFP-IR circumferential scar extent was slightly underestimated (total score 3726). Details are shown in Fig. 3a.

On all multi-slice sequences, the visual allocation of LGE within the myocardial wall (subendocardial, subepicardial, intramural, transmural) showed good matching with FLASH-PSIR for the chronic infarction and inflammatory heart disease group while for inflammatory heart disease there was a higher number of visually transmural LGE areas on bSSFP-PSIR versus FLASH-PSIR (14% versus 10% segments with transmural LGE, see Fig. 3b).

### Quantitative LGE analysis

**Fig. 4:**
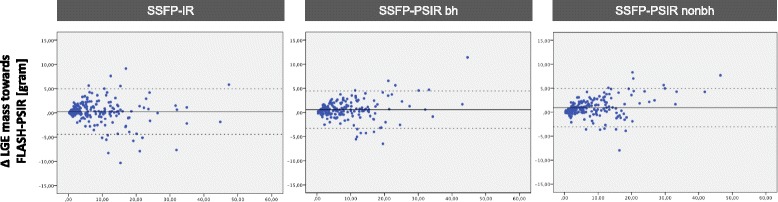
Bland-Altman plots of LGE mass. Blue dots represent mean LGE size (x-axis) versus delta LGE mass towards FLASH-PSIR (y-axis) in gram for each LGE sequence

There were no significant differences in mean LGE size between reference standard FLASH-PSIR and multi-slice sequences independent from LGE origin (Table 4). However, Bland-Altman analysis showed that on bSSFP-PSIR LGE size showed a non-significant trend to be smaller in all study groups compared to reference standard - mean difference in LGE size towards reference standard being 0.58 ± 1.99 g on bSSFP-PSIR with breathhold, 0.96 ± 2.03 g on bSSFP-PSIR with free breathing and 0.26 ± 2.4 g on bSSFP-IR (see Fig. 4 for Bland-Altman plots).

**Table 4:** Quantitative Assessment - LGE size

	All groups	Chronic infarction	HCM	Inflammatory heart disease	
FLASH-PSIR	8.96 ± 10.64 g	7.47 ± 6.65 g	15.42 ± 20.00 g	9.39 ± 10.28 g	
SSFP-IR	8.69 ± 10.75 g	7.26 ± 7.03 g	15.31 ± 20.02 g	8.67 ± 9.66 g	p > 0.05
SSFP-PSIR	9.05 ± 10.84 g	7.68 ± 7.18 g	15.51 ± 20.31 g	8.89 ± 9.30 g	p > 0.05
SSFP-PSIR nonbh	8.85 ± 10.71 g	7.41 ± 6.91 g	15.38 ± 19.96 g	8.97 ± 9.94 g	p > 0.05

Values represent mean LGE size in gram. *P* values for each multi-slice sequence compared to FLASH-PSIR in all study groups

The presence or absence of breathhold during LGE imaging using bSSFP-PSIR had no impact on LGE size for any disease entity (Table 4).

Intraobserver agreement (Pearson coefficient) on LGE size was > 0.95 for all sequences. Interobserver agreement was 0.92 for bSSFP-PSIR under free breathing and 0.88 for all other sequences.

In patients with arrhythmia during image acquisition mean LGE size did not differ in any multi-slice sequence, with bSSFP-IR 7.6 ± 6.1 g and bSSFP-PSIR 7.7 ± 5.6 g under breathhold and 7.4 ± 5.6 g under free breathing. Reference standard FLASH-PSIR sequence was not evaluated in arrhythmic patients due to mostly non-diagnostic image quality.

## Discussion

The present study compared for the first time a reference standard segmented (FLASH-PSIR) with two multi-slice LGE sequences (bSSFP-IR and bSSFP-PSIR) in 298 patients with ischemic and non-ischemic cardiomyopathies.

Our key findings were: Firstly, image quality and CNR were highest on multi-slice bSSFP-PSIR with and without breathhold. Secondly, acquisition time is relevantly shorter on any multi-slice sequence compared to reference standard. Thirdly, visual detection of LGE and visual assessment of LGE extent was consistently very good and equivalent in all sequences. Fourthly, quantification showed no significant difference in LGE size for any multi-slice sequence. Fifthly, in patients with arrhythmia all multi-slice sequences generated good image quality and consistent LGE quantification results, whereas the reference standard provided non-diagnostic image quality in half of all exams. Finally, acquisition of bSSFP-PSIR under free breathing or under breathhold had no impact on LGE detection and quantification. Results were independent of the cause of LGE from ischemic or non-ischemic etiology.

The assessment of myocardial fibrosis has enormous diagnostic and prognostic impact in ischemic and non-ischemic cardiomyopathy [5–7]. Over the last decade many clinical studies have paved the way for CMR to be integrated into a variety of cardiologic, radiologic and other clinical guidelines [9]. The role of LGE in detection of myocardial fibrosis remains unequivocally important despite the development of new parametric mapping techniques, which play an increasing role especially in detection of diffuse fibrosis [28, 29].

Our study demonstrates that bSSFP-PSIR and bSSFP-IR multi-slice LGE sequences provide excellent alternatives to segmented FLASH-PSIR in routine CMR protocols. We showed that not only for ischemic LGE lesions but also for more diffuse lesions in inflammatory heart disease or HCM multi-slice sequences are sufficient to visualize fibrosis and – when quantified in size – show equivalent results compared to the reference standard. The equivalence of multi-slice LGE sequences to segmented sequences has previously been shown in studies for either HCM, ischemic or inflammatory heart disease [18, 19, 30]. However, these studies each used different sequences, smaller patient groups and mostly looked at single disease entities.

The superiority of multi-slice over segmented PSIR sequences in regard to image quality and CNR is in line with other publications [18, 19]. This is attributable to the reduction of motion artifacts and artifacts due to arrhythmia. CNR also depends on the amount of gadolinium-based contrast media in the myocardium, which is influenced by amount and molarity of contrast agent, distribution volume and hemodynamics. As we strictly dosed gadolinium to body weight and tested sequences in a random order, effects on results should be neglectable.

We have also seen variations in CNR between the different disease entities. Since CNR is dependent on the voxel composition of fibrotic tissue, LGE in chronic infarction with more compact fibrosis is expected to result in higher CNR values than LGE in more diffusely fibrotic tissue such as in HCM and inflammatory heart disease.

On bSSFP-PSIR sequence visual assessment revealed slightly larger scar transmurality as compared to the reference standard. We believe that visual assessment of bSSFP-PSIR images is impacted by its comparably higher CNR values which may lead to subjectively higher transmurality of scars.

In two patients, small LGE lesions detectable with the reference standard sequence, were not detected with multi-slice bSSFP-PSIR but, nevertheless, could be visualized with bSSFP-IR. Note that for these two patients LGE amount was less than one gram, which suggests that partial volume effects or shifted slice position due to heavy respiratory motion may have caused the missed lesion. However, it cannot be safely excluded that very small LGE lesions may be missed with multi-slice bSSFP-PSIR due to its different matrix size as compared to the reference standard.

It has been shown that even a small amount of LGE has prognostic implications in cardiomyopathies [31–34]. In case of inflammatory heart disease missed small subepicardial LGE may even impact diagnosis [35], as Lake Louise criteria define myocarditis as two out of three parameters, LGE being one of them [36].

Our results suggest that in patients with known or suspected myocardial infarction bSSFP-PSIR or bSSFP-IR multi-slice sequences can be primarily utilized for LGE detection. In case of an unknown cardiomyopathy or for assessment of HCM and inflammatory heart disease segmented FLASH-PSIR images should be used in scenarios of stable sinus rhythm and sufficient breathhold capabilities. For patients with arrhythmia and/or insufficient breathhold capabilities at the time of CMR scan we showed that segmented sequences fail to provide sufficient image quality. This is in line with previous studies [37, 38]. In these patients we suggest to primarily use multi-slice sequences such as bSSFP-PSIR and/or bSSFP-IR.

In this study two different contrast media were used; gadoteridol in CAD and HCM patients and gadopentate for inflammatory heart disease. The reason for use of gadopentate was established normal values for relative enhancement sequences, which were acquired in these patients independently from this study. However, there is good evidence that relaxivity and contrast enhancement are nearly identical for both agents so that impact on results should be neglectable. [39]

In our study we explicitly did not exclude patients with arrhythmia. We demonstrated good to excellent image quality and equivalent amount of LGE quantification with all multi-slice sequences. There is no gold standard for LGE detection and quantification in arrhythmic patients. Hence, we cannot definitely state that results are perfectly correct using multi-slice sequences. Still, due to the consistently high image quality scores and – as compared to patients with sinus rhythm – similar CNR values we feel confident that usage of any multi-slice sequence is superior to attempts of segmented image acquisition and shortens scan protocols significantly in these patients.

Interestingly, presence or absence of breathhold during image acquisition on bSSFP-PSIR did not affect detection or quantification of LGE across all study groups. While there must be minimal slice shifting due to respiratory motion on acquisition of an entire LV short axis package within approximately 20 s of acquisition time we could show in a large number of patients that this has no statistically significant effect on diagnostic value. Lower numbers of breathhold cycles may also positively affect patient comfort and may be considered in all patients when SSFP-PSIR sequence is used.

Alternative methods for LGE assessment include 3D sequences, which have been shown to also accurately visualize fibrosis and scarring [40, 41]. These 3D sequences have the advantage of potentially higher spatial resolution, especially in the vertical axis, and the possibility of free movement through the ventricular myocardium. On the other hand, 3D sequences typically require a relatively long acquisition time. This necessitates a continuous adaptation of the optimal myocardial inversion time, which may impair image quality and CNR. Implementation of 3D LGE sequences with dynamic inversion time adjustments may help to overcome this obstacle [42].

In another recent study dark blood PSIR imaging was published using T2 preparation pulses for improved visualization of fibrosis close to the adjacent LV blood pool in 30 patients with subendocardial infarction [43]. This promising technique also included motion correction for acquisition under free breathing but needs to be validated across myocardial disease entities in larger studies.

## Conclusions

LGE sequences are a mandatory part of most CMR protocols in ischemic and non-ischemic cardiomyopathy [8]. The broader spread of CMR in clinical routine has several implications: demand for CMR access increases and there is a continuous need for fast scanning protocols [14]. While segmented LGE sequences may give excellent image quality under stable sinus rhythm and sufficient breathhold the issue of time investment prevails. We demonstrated equivalence of multi-slice LGE sequences (bSSFP-IR and bSSFP-PSIR) and segmented FLASH-PSIR sequence in a large number of patients with ischemic and non-ischemic cardiac disease.

For that reason we suggest further strengthening the role of multi-slice sequences in routine CMR protocols whenever it is reliable to use.

### Limitations

In spite of the large patient number in this study, all patients were scanned and analyzed in a single CMR center. Male gender was overrepresented in all study groups. Comparability of LGE sequences maybe impacted by different matrix size and, hence, different in-plane resolution used for each sequence. This may also affect SNR and CNR as well as assessment of LGE-positive areas.

## Abbreviations

2D: Two-dimensional
AHA: American Heart Association
BMI: Body mass index
bSSFP: Balanced steady-state free precession
CMR: Cardiovascular magnetic resonance
CNR: Contrast-to-noise ratio
FLASH: Fast low angle shot
HCM: Hypertrophic cardiomyopathy
HR: Heart rate
IR: Inversion recovery
LGE: Late gadolinium enhancement
LV: Left ventricle/left ventricular
LVEDV: Left ventricular end-diastolic volume
LVEDV-I: Left ventricular end-diastolic volume index
LVEF: Left ventricular ejection fraction
LVM: Left ventricular mass
LVM-I: Left ventricular mass index
MR: Magnetic resonance
nbh: Non-breathhold (free breathing)
PSIR: Phase-sensitive inversion recovery
ROI: Region-of-interest
RV: Right ventricle/right ventricular
SD: Standard deviation
SNR: Signal-to-noise ratio
SR: Sinus rhythm
STRM: Semi-automated signal threshold versus reference mean
SV-I: Stroke volume index
TE: Echo time
TI: Inversion time
